# Clinical Progression Rates by CD4 Cell Category Before and After the Initiation of Combination Antiretroviral Therapy (cART)

**DOI:** 10.2174/1874613600802010003

**Published:** 2008-02-12

**Authors:** Marguerite Guiguet, Kholoud Porter, Andrew Phillips, Dominique Costagliola, Abdel Babiker

**Affiliations:** 1 INSERM U720, Paris, France; 2 UPMC Paris 06 UMR S720, Paris, France; 3 MRC Clinical Trials Unit, London, UK; 4 Royal Free & University College Medical School, London, UK

**Keywords:** Disease progression, CD4, cART, event rates, prognostic value.

## Abstract

**Objective:**

Rates of AIDS defining event (ADE), serious ADE and death by CD4 and HIV RNA categories before and after combination antiretroviral therapy (cART) initiation are lacking for high CD4 counts.

**Methods:**

Event rates were estimated within CD4 cell strata using a Poisson regression model adjusting for sex, exposure category, age, and current HIV RNA (<4, 4-4.99, ≥5 log copies/ml), and including an interaction term between the CD4 cell count and cART indicator.

**Results:**

7317 and 6376 persons contributed to "naïve " and "cART " groups respectively, of whom 3911 contributed to both. At the same CD4 level, the risk of ADE was nearly 2 fold higher during naive follow-up compared to cART for CD4 <500 cells/mm^3^. However, after adjustment for current HIV RNA, the risk of ADE became similar for both groups except for CD4 count <200 cells/mm^3^ when it is 35% (6-72%) higher for naives. The same results were observed for the risk of serious ADE. There was no evidence of a difference in risk of death between naive and cART follow-up at specific CD4 categories even after adjustment for HIV RNA.

**Conclusion:**

Within CD4 cell strata above 200 cells/mm^3^, the risk of ADE before ART initiation is higher than it is following cART initiation.

## INTRODUCTION

The SMART trial recently closed recruitment after interim analyses demonstrated that persons undergoing a treatment interruption once their CD4 cell count reached >350 cells/mm^3^, restarting once it fell to <250 cells/mm^3^, experienced significantly worse outcomes compared to those randomised to continuous therapy [1]. These CD4 thresholds were chosen to provide a safety margin above 200 cells/mm^3^, the level at which current treatment guidelines recommend initiation of therapy. These disappointing results have rekindled the debate on whether the time is ripe for a “when to start” trial to evaluate benefits and risks from initiating antiretroviral therapy (ART) at higher CD4 counts than the current 200-350 cells/mm^3^ level at which it is considered safe to do so [2].

However, there are few published data to inform such a trial design [3]. We used data from CASCADE, a large collaboration of seroconverter cohorts with CD4 and viral load measurements available prior to the initiation of therapy and once it is initiated to provide estimates of rates of AIDS, and death, at different CD4 categories for persons naïve to therapy as well as those who started combination ART (cART).

We also use the derived rates to assess the extent to which any differences in risk before and after cART initiation could be explained by the effect of cART on HIV RNA level.

## PATIENTS AND METHODS

The CASCADE collaboration includes 23 cohorts of persons with well-estimated dates of HIV seroconversion and has been described in detail elsewhere [4]. After exclusion of patients who started ART in the first 6 months following seroconversion, follow-up was categorised as either "naïve", including all follow-up while patients were AIDS-free and antiretroviral naive individuals at their first CD4 cell count after 1 January 1997, and "cART", comprising all follow-up of patients once a combination of antiretroviral therapy was initiated after 1 January 1997, with at least 3 antiretroviral (ARV) drugs, or 2 boosted Protease Inhibitors (PI), or one boosted PI and one Non-nucleoside Reverse Transcriptase Inhibitor (NNRTI). A given individual could contribute follow up in both categories. Follow-up under non-cART regimens was ignored. For each follow-up category, the baseline was defined as the first visit for which an individual's follow-up qualified for inclusion into that category and until the last visit in that category. For "cART", CD4 cell count and HIV RNA at baseline were the closest values before cART initiation and up to a maximum of 6 months prior to initiation.

The following clinical events were studied: (i) new AIDS-defining event (ADE), (ii) new serious ADE (all AIDS events except for recurrent bacterial pneumonia, oesophageal candidiasis, reccurent herpes simplex, pulmonary and extrapulmonary tuberculosis, and unspecified events), (iii) death, and two composite end-points: (iv) new ADE or death, (v) new serious ADE or death. Patients who died from AIDS without a previous AIDS diagnosis were classified as ADE progressors. For patients who were not AIDS-free at inclusion in the study group, progression was defined by the occurrence of the first new clinical event. CD4 cell counts were measured with a median periodicity of 98 days, and 91 days, during the naïve and cART follow-up periods, respectively. CD4 cell counts were modelled using linear interpolation between two measurements. The viral load was determined with a median frequency of 105 days, and 91 days, during the naïve, and cART, follow-up, respectively. CD4 cell count was categorized in five specific strata (<200, 200-350, 350-500, 500-650 and ≥650 cell/mm^3^), and HIV RNA in three levels (<4, 4-4.99, ≥5 log copies/ml) [3].

For each group of follow-up, incidence of each outcome was estimated within each CD4 cell count stratum. In order to test whether, for a given CD4 stratum, the risk of an event differs between ART-naïve patients and those who had started cART, we included “cART” as an indicator variable in a Poisson regression model with an interaction term between the CD4 cell count and cART. We adjusted for the effect of the following potential confounders: sex and exposure category (as a combined variable), and age. A separate model further adjusted for current HIV RNA. In assessing whether the risk of an event differed between those ART-naïve and those who had started cART within each stratum of CD4 count, an interaction term between the CD4 cell count and cART indicator was significant whether the risk of ADE was modelled excluding HIV RNA (p=0.002) or including it (p=0.03). This interaction term was, therefore, included in both models. Statistical analyses were performed using SAS software package version 9.1 (SAS Institute, Cary, North Carolina, USA).

## RESULTS

A total of 7317 patients contributed 12 297 Person-Years (PY) of antiretroviral-naïve follow-up with median baseline CD4 and HIV RNA of 477 cells/mm^3^ and 4.5 log copies/ml respectively. After cART initiation, 6376 patients, of whom 3690 (58%) were pre-treated, contributed 28 864 PY. Of these 6376 patients, 3911 (61%) were also followed-up as naives, and hence contributed person years to both categories. Median baseline CD4 and HIV RNA for the patients on cART was 310 cells/mm^3^ and 4.5 log copies/ml respectively (Table **1**). The first cART prescription was PI-containing for 57%, NNRTI-containing for 30%, Nucleoside Reverse Transcriptase Inhibitor (NRTI) only for 10%, and another combination for 3%. Six months after cART initiation (3 to 9 months), 73% of previously ART-naive patients experienced an increase of >50 cells/mm^3^ from baseline and 79% achieved HIV RNA <500 copies/ml. The corresponding values for pre-treated patients were 56% and 59% respectively.

Overall, 227 and 146 ADE and serious ADE, and 100 deaths were observed for the naïve follow-up with a corresponding number of events of 498, 335, and 360 during cART follow-up, respectively. Event rates were higher with lower CD4 cell counts (Table **2**). For ART-naïve individuals, ADE rates were markedly higher in those with CD4 count below 500 cells/mm^3^ compared with higher CD4, varying from 0.5 event/100 PY (95% Confidence Interval [CI] 0.2-0.7) for individuals with CD4 500-650 cells/mm^3^ and rising to 1.2 (0.8-1.5), 2.6 (1.8-3.2), and 21.8 (17.3-26.2) events/100 PY, respectively, at CD4 350-500, 200-350, and <200 cells/mm^3^. The same trend was observed for serious ADE rates. For those who initiated cART, ADE and serious ADE rates were generally <1 event/100 PY in CD4 categories >350 cells/mm^3^. The risk of death remained at <1 event/100 PY for CD4 >200 cells/mm^3^. The risk of ADE or death overall was 2.5/100PY, and 2.6/100PY, for naïve, and cART follow-up respectively, and the respective risk of serious ADE or death was 1.9/100PY, and 2.1/100PY.

Without adjustment for current HIV RNA, the risk of ADE was nearly 2 fold higher for ART-naive individuals compared to those who started cART for the CD4 count categories below 500 cells/mm^3^, with risk augmentation of 58% (95%CI, 4- 140), 78% (95%CI, 24-156), and 85% ( 95%CI, 46- 135) for CD4 350-500, 200-350, and <200 cells/mm^3^ respectively (Model 1- Table **3**). The risk of ADE became similar for naives and for those who started cART after adjustment for current HIV RNA, except for when the CD4 count was below 200 cells/mm^3^ when the risk of ADE remained significantly higher for naive individuals (Model 2- Table **3**). The data provide evidence that the risk of serious ADE was significantly higher for naives compared to those who started cART, only for CD4 <350/mm^3^, before adjusting for current HIV RNA (RR and 95%CI= 1.68, 1.07-2.62 and 2.06, 1.57- 2.71 respectively for CD4 strata 200-350 and <200 cells/mm^3^). After adjustment for current HIV RNA, the risk of serious ADE within CD4 categories appeared similar for naives and those who started cART, except for when the CD4 dropped below 200/mm^3^, as with the risk of ADE. In contrast, for fixed CD4 level, there was no association between treatment and risk of death irrespective of adjustment for HIV RNA. When the analyses were restricted to AIDS-free patients at cART initiation, the same results were observed (not shown). Fig. (**1**) shows the rates of ADE, serious ADE, and death estimated using Poisson regression. The differences in rates of clinical progression between the ART-naïve and cART follow-up, which were observed at CD4 counts 200-500 cells/mm^3^, disappeared once we adjusted for current HIV RNA. This suggests that CD4 and HIV RNA had the same prognostic values for naives as for those who started cART, except for CD4 <200 cells/mm^3^ where the independent effect of cART was more pronounced.

## DISCUSSION

Events rates are extremely high within the lowest CD4 cell stratum (<200 cells/mm^3^), as reported by a number of studies [5-8] and fall to 0.5-1 event per 100 PY in those with current CD4 count >350 cells/mm^3^. However, the risk of ADE was nearly halved after cART initiation compared to naïve follow-up when the CD4 count was below 500 cells/mm^3^. If it can be shown through a randomized controlled trial (RCT) that ART is indicated at these higher levels, this has cost and operational implications for HIV treatment and care programmes in developing countries. This is not only because of the additional numbers who would be eligible for cART, but also because there would likely be no need for CD4 testing to evaluate whether an HIV infected person is eligible for treatment.

It is not surprising that at CD4 >200 cells/mm^3^ and after adjustment for current HIV RNA, the relative risk of an event was similar during cART follow-up and naïve follow-up because HIV RNA is a surrogate for being on cART. This finding is in contrast to that reported from the Frankfurt HIV Outpatient Clinic cohort at the beginning of cART era of lower event rates for patients at the same CD4 cell count and viral load levels receiving a PI-containing regimen compared to those not on therapy [9]. The reason for this is not clear, but it is of note that much higher event rates were reported by that study compared to those observed in our own study, both for treated and naïve follow-up. However, after adjusting on HIV RNA, an independent effect of cART was still observed in our study with 26% of risk reduction at low CD4 cell count.

We noted relatively high death rates among ART-naïve patients with CD4 >350 cells/mm^3^. These appear to be due to an excess of suicides, accidental deaths and deaths due to substance abuse within those CD4 strata as these causes accounted for 53% (8/15) of deaths at CD4 >650 cells/mm^3^, and for 66% (2/3) and 44% (8/18) of deaths in persons with CD4 500-650 and 350-500 cells/mm^3^ respectively.

Our study has a number of limitations. Firstly, included in our cART category are persons who were naïve at the time of initiation as well as pre-treated individuals. Although this may tend to increase event rates, our observed rates were, in fact, lower than those reported by the UK Collaborative HIV Cohort Study (CHIC) [3]. It is important to note, however, that in CHIC, the ADE and serious ADE rates, in fact, included death. The exclusion of pre-treated individuals did not, in any case, have an effect on the evaluation of the prognostic value of CD4 and HIV RNA in cART-treated compared to naïve follow-up (data not shown). In addition, 28%, and 16%, of deaths among naive and treated individuals, respectively, had no cause recorded preventing us from evaluating whether the prognostic value of CD4 and HIV RNA is more pronounced for specific causes. Finally, individuals who happen to be on cART at a given CD4 count and HIV RNA levels are different from those who are not. Although our analyses adjust for the potential effects of age and exposure category as confounders, given the observational nature of our study, unmeasured confounders remain which make it difficult to compare appropriately event rates for those on treatment with those who remain off it. In an attempt to limit the effect of this bias, we assigned events diagnosed in the first week of treatment initiation to the naïve category. Earlier access to therapy, and better immunological and virological responses have been observed among homosexual men compared to other groups, particularly to injecting drug users (IDU) [10]. A difference in clinical benefit of treatment according to patient characteristics, such the transmission group, has also been reported [8]. A number of factors, such as use of health care, lifestyles, level of adherence to therapy, as well as the regimen itself, are important in determining event rates for those initiating cART and could explain differences in event rates between studies. These factors will also impact on an evaluation of differences in event rates between treated and untreated individuals who are at similar CD4 and HIV RNA levels.

## CONCLUSIONS

Comparing clinical event rates between ART-naives and cART-treated follow-up within CD4 cell strata above 200 cells/mm^3^, the risk of AIDS before ART initiation is higher than the risk following cART initiation. However, whether cART should be initiated at higher CD4 levels than is currently recommended in the guidelines, can only be evaluated through a randomised controlled trial.

## Figures and Tables

**Figures (1):**
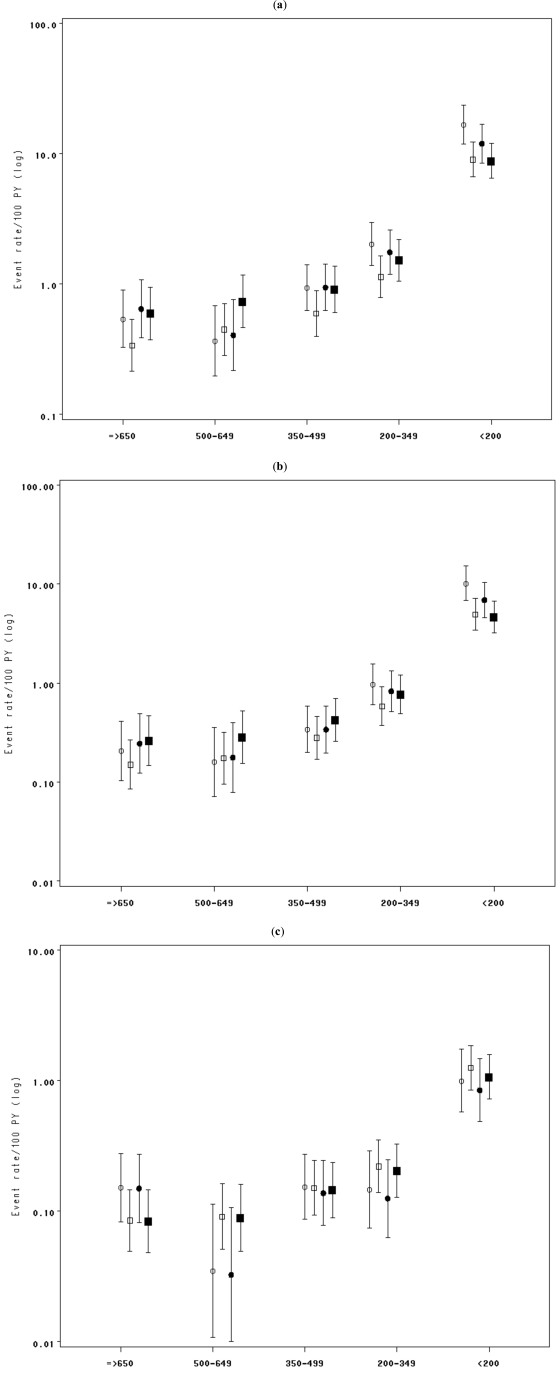
Estimates of rates and 95%CI of (a) AIDS-defining event (ADE), (b) serious ADE, (c) death, before (circle) and after (square) initiation of combination anti-retroviral therapy (cART) using Poisson regression model adjusting on age and exposure category/sex (hollow symbol), or adjusting on age, exposure category/sex and current HIV RNA strata (solid symbol), with an interaction term between cART indicator and CD4 strata

**Table 1:** Characteristics of Patients Before and After the Initiation of Combination Antiretroviral Therapy (cART)

	ART-Naïve	cART
Baseline*		
Number of patients	7317	6376
Age (yrs), median (IQR°)	33 (28-39)	35 (30-41)
Exposure category/sex (%)		
Sex between men	4074 (56)	3269 (51)
Male: injecting drug use	685 (9)	703 (11)
Female: injecting drug use	348 (5)	396 (6)
Male: sex between men and women	698 (9)	616 (10)
Female: sex between men and women	1180 (16)	1059 (17)
Other/not known	332 (5)	333 (5)
Time (yrs) from seroconversion to baseline, median (IQR)	1.1 (0.5-3.3)	4.6 (1.8-7.9)
CD4 (cells/mm3), median (IQR)	477 (340-648)	310 (201-447)
HIV RNA (log10 copies/ml), median (IQR)	4.5 (3.9-5.1)	4.6 (3.8-5.1)
Follow-up		
Number of Person-Years	12 297	28 864
Duration of follow-up (yrs), median (IQR)	0.8 (0.2-2.5)	4.6 (2.1-6.9)
Number of CD4 measurements, median (IQR)	3 (2-7)	13 (6-23)
Number of HIV RNA measurements, median (IQR)	3 (1-6)	12 (5-22)
Cumulative time spent in CD4 strata, years (%)		
>650	2981 (24)	7357 (25)
500-650	2643 (21)	5406 (19)
350-500	3520 (29)	6403 (22)
200-350	2040 (17)	5004 (17)
<200	458 (4)	2654 (9)
missing	653 (5)	2039 (7)

* 3911 patients contributed to both categories of follow-up. Baseline characteristics are measured at the first visit for "ART-naïve" and at treatment initiation for "cART" ^°^IQR : interquartile range

**Table 2:** Crude Incidence Rates (95% Confidence Intervals) Per 100 Person-Years (PY), Number with Event and PY of Follow-Up in Brackets [], According to Current CD4 Cell Count (Cells/mm3) Before and After the Initiation of Combination Antiretroviral Therapy (cART)

	CD4	ART-Naïves	cART
ADE^°^	>650	0.7 (0.4-1.0)	0.4 (0.3-0.6)
		[20/2967]	[31/7207]
	500-649	0.5 (0.2-0.7)	0.6 (0.4-0.8)
		[12/2633]	[30/5258]
	350-499	1.2 (0.8-1.5)	0.7 (0.5-1.0)
		[41/3494]	[47/6201]
	200-349	2.6 (1.8-3.2)	1.4 (1.1-1.8)
		[51/1997]	[69/4734]
	<200	21.8 (17.3-26.2)	11.7 (10.3-13.2)
		[92/422]	[260/2214]
	Missing	1.7 (0.7-2.8)	3.1 (2.3-3.8)
		[11/625]	[61/1980]
Serious ADE	>650	0.3 (0.1-0.5)	0.3 (0.2-0.4)
		[10/2977]	[19/7266]
	500-649	0.3 (0.1-0.5)	0.3 (0.2-0.5)
		[7/2640]	[16/5321]
	350-499	0.6 (0.3-0.8)	0.5 (0.3-0.6)
		[20/3512]	[30/6280]
	200-349	1.6 (1.1-2.2)	1.0 (0.7-1.3)
		[33/2020]	[47/4831]
	<200	16.1 (12.3-19.9)	7.8 (6.7-8.9)
		[71/440]	[185/2366]
	Missing	0.8 (0.1-1.5)	1.9 (1.3-2.8)
		[5/639]	[38/2001]
Death	>650	0.5 (0.2-0.8)	0.3 (0.2-0.5)
		[15/2981]	[24/7357]
	500-649	0.1 (0.0-0.2)	0.4 (0.2-0.5)
		[3/2643]	[19/5406]
	350-499	0.5 (0.3-0.7)	0.6 (0.4-0.8)
		[18/3520]	[38/6403]
	200-349	0.5 (0.2-0.8)	0.9 (0.7-1.2)
		[11/2040]	[47/5004]
	<200	4.6 (2.6-6.5)	5.9 (5.0-6.9)
		[21/458]	[158/2654]
	Missing	4.9 (3.2-6.6)	3.6 (2.8-4.5)
		[32/653]	[74/2039]
ADE/death	>650	1.1 (0.7-1.5)	0.7 (0.5-0.9)
		[33/2967]	[52/7207]
	500-649	0.6 (0.3-0.8)	0.9 (0.6-1.1)
		[15/2633]	[46/5258]
	350-499	1.6 (1.2-2.0)	1.3 (1.0-1.6)
		[56/3494]	[82/6201]
	200-349	3.1 (2.3-3.8)	2.2 (1.8-2.6)
		[61/1997]	[105/4734]
	<200	23.2 (18.6-27.8)	14.6 (13.0-16.2)
		[98/422]	[324/2214]
	Missing	6.2 (4.3-8.2)	5.2 (4.2-6.2)
		[39/625]	[104/1980]
Serious ADE/death	>650	0.8 (0.5-1.1)	0.6 (0.4-0.8)
		[23/2977]	[42/7266]
	500-649	0.4 (0.1-0.6)	0.6 (0.4-0.8)
		[10/2640]	[32/5321]
	350-499	1.1 (0.7-1.4)	1.0 (0.8-1.3)
		[38/3512]	[65/6280]
	200-349	2.1 (1.5-2.8)	1.8 (1.4-2.2)
		[43/2020]	[86/4831]
	<200	19.1 (15.0-23.2)	11.5 (10.2-12.9)
		[84/440]	[273/2366]
	Missing	5.5 (3.7-7.3)	4.6 (3.7-5.6)
		[35/639]	[93/2001]

^°^ADE : AIDS defining event.

**Table 3:** Effect of Combination Antiretroviral Therapy (cART) on Event Rates –AIDS Defining Event (ADE), Serious ADE, or Death- Within CD4 Cell Strata: Relative Rate (95% Confidence Interval) of Clinical Progression for ART-Naïve Follow-Up Compared to cART Follow-Up

	CD4 Strata (cells /mm^3^)	Model 1	Model 2
		RR (95%CI)	RR (95%CI)
ADE	>650	1.60 (0.91-2.81)	1.08 (0.62-1.92)
	500-650	0.82 (0.42-1.60)	0.55 (0.28-1.09)
	350-500	1.58 (1.04-2.40)	1.04 (0.68-1.60)
	200-350	1.78 (1.24-2.56)	1.15 (0.79-1.66)
	<200	1.85 (1.46-2.35)	1.35 (1.06-1.72)
Serious ADE	>650	1.37 (0.64-2.95)	0.93 (0.43-2.03)
	500-650	0.91 (0.38-2.22)	0.62 (0.25-1.53)
	350-500	1.21 (0.69-2.14)	0.80 (0.45-1.42)
	200-350	1.68 (1.07-2.62)	1.07 (0.68-1.69)
	<200	2.06 (1.57-2.71)	1.48 (1.12-1.95)
Death	>650	1.78 (0.93-3.41)	1.79 (0.93-3.43)
	500-650	0.38 (0.11-1.30)	0.37 (0.11-1.25)
	350-500	1.02 (0.58-1.79)	0.95 (0.53-1.68)
	200-350	0.67 (0.35-1.28)	0.61 (0.31-1.19)
	<200	0.80 (0.51-1.26)	0.79 (0.50-1.25)

Estimates of event rates using Poisson regression model adjusting on age and exposure category/sex (model 1), or age, exposure category/sex and current HIV RNA strata (model 2), with an interaction term between cART indicator and CD4 strata.

## APPENDIX-CASCADE

### Steering Committee:

Julia Del Amo (Chair), Laurence Meyer (Vice Chair), Heiner Bucher, Geneviève Chêne, Deenan Pillay, Maria Prins, Magda Rosinska, Caroline Sabin, Giota Touloumi **Co-ordinating Centre:** Kholoud Porter (Project Leader), Krishnan Bhaskaran (Scientific Co-ordinator), Sarah Walker, Abdel Babiker, Janet Darbyshire **Clinical Advisory Board:** Heiner Bucher, Andrea de Luca, Martin Fisher, Cécile Goujard, Roberto Muga

### Collaborators:

**Australia **Sydney AIDS Prospective Study and Sydney Primary HIV Infection cohort (John Kaldor, Tony Kelleher, Tim Ramacciotti, Linda Gelgor, David Cooper, Don Smith);** Canada **South Alberta clinic (John Gill);** Denmark** Copenhagen HIV Seroconverter Cohort (Louise Bruun Jørgensen, Claus Nielsen, Court Pedersen);** Estonia **Tartu Ülikool (Irja Lutsar);** France **Aquitaine cohort (Geneviève Chêne, Francois Dabis, Rodolphe Thiebaut, Bernard Masquelier), French Hospital Database (Dominique Costagliola, Emilie Lanoy, Marguerite Guiguet),****Lyon Primary Infection cohort (Philippe Vanhems),****SEROCO cohort (Laurence Meyer, Faroudy Boufassa);** Germany **German cohort****(Osamah Hamouda, Claudia Kucherer);** Greece **Greek Haemophilia cohort****(Giota Touloumi, Nikos Pantazis, Angelos Hatzakis, Dimitrios Paraskevis, Anastasia Karafoulidou); **Italy** Italian Seroconversion Study (Giovanni Rezza, Maria Dorrucci, Benedetta Longo, Claudia Balotta);** Netherlands **Amsterdam Cohort Studies among homosexual men and drug users (Maria Prins, Liselotte van Asten, Akke van der Bij, Ronald Geskus, Roel Coutinho);** Norway **Oslo and Ulleval Hospital cohorts (Mette Sannes, Oddbjorn Brubakk, Anne Eskild, Johan N Bruun);** Poland **National Institute of Hygiene (Magdalena Rosinska);** Portugal **Universidade Nova de Lisboa (Ricardo Camacho);** Russia** Pasteur Institute (Tatyana Smolskaya);** Spain** Badalona IDU hospital cohort (Roberto Muga), Barcelona IDU Cohort (Patricia Garcia de Olalla),****Madrid cohort (Julia Del Amo, Jorge del Romero),****Valencia IDU cohort (Santiago Pérez-Hoyos, Ildefonso Hernandez Aguado);** Switzerland **Swiss HIV cohort (Heiner Bucher, Martin Rickenbach, Patrick Francioli);** Ukraine** Perinatal Prevention of AIDS Initiative (Ruslan Malyuta);** United Kingdom** Edinburgh Hospital cohort (Ray Brettle), Health Protection Agency (Valerie Delpech, Sam Lattimore, Gary Murphy, John Parry, Noel Gill), Royal Free haemophilia cohort (Caroline Sabin, Christine Lee),****UK Register of HIV Seroconverters (Kholoud Porter,****Anne Johnson, Andrew Phillips, Abdel Babiker, Janet Darbyshire, Valerie Delpech),****University College London (Deenan Pillay), University of Oxford (Harold Jaffe).
